# A Walk-In Clinic for Newly Arrived Mentally Burdened Refugees: The Patient Perspective

**DOI:** 10.3390/ijerph18052275

**Published:** 2021-02-25

**Authors:** Catharina Zehetmair, Valentina Zeyher, Anna Cranz, Beate Ditzen, Sabine C. Herpertz, Rupert Maria Kohl, Christoph Nikendei

**Affiliations:** 1 Center for Psychosocial Medicine, Department for General Internal Medicine and Psychosomatics, Heidelberg University Hospital, 69115 Heidelberg, Germany; valentina.zeyher@gmail.com (V.Z.); anna.cranz@med.uni-heidelberg.de (A.C.); christoph.nikendei@med.uni-heidelberg.de (C.N.); 2 Center for Psychosocial Medicine, Institute of Medical Psychology, Heidelberg University Hospital, 69115 Heidelberg, Germany; beate.ditzen@med.uni-heidelberg.de (B.D.); RupertMaria.Kohl@med.uni-heidelberg.de (R.M.K.); 3 Center for Psychosocial Medicine, Department of General Psychiatry, Heidelberg University Hospital, 69115 Heidelberg, Germany; sabine.herpertz@med.uni-heidelberg.de

**Keywords:** refugees, mental burden, psychosocial support, mental health service, qualitative analyses

## Abstract

Providing refugees with psychosocial support is particularly important considering the high level of mental health problems prevalent in this population. A psychosocial walk-in clinic operating within a state reception and registration center in Germany has been supporting mentally burdened refugees since 2016. This study focused on patients’ perspectives on their mental health burden, the psychosocial walk-in clinic, and future help seeking. We conducted interviews with *n* = 22 refugees attending the walk-in clinic from March to May 2019. Qualitative analysis focused on the following four topics: (1) mental burden from the patients’ perspective, (2) access to the psychosocial walk-in clinic, (3) perception of counseling sessions, and (4) perception of follow-up treatment. The results show that the majority of interviewees were burdened by psychological and somatic complaints, mostly attributed to past experiences and post-migratory stress. Therapeutic counseling and psychiatric medication were found to be particularly helpful. Most of the participants felt motivated to seek further psychosocial support. Key barriers to seeking psychosocial help included shame, fear of stigma, and lack of information. Overall, the psychosocial walk-in clinic is a highly valued support service for newly arrived refugees with mental health issues.

## 1. Introduction

Around 37,000 people a day are forced to flee their homes according to the United Nations High Commissioner for Refugees (UNHCR) [1]. The majority of refugees are internally displaced persons or refugees seeking relief close to home in neighboring countries. About one-third of global refugees flee to neighboring, less developed, or least developed countries. In 2019, about 200,000 refugees sought safety in Europe by crossing the Mediterranean Sea [2]. Refugees are a high-risk population for mental health problems as a result of stressful and traumatizing events and circumstances in their country of origin as well as during and after their flight. Prevalence rates of up to 40% for any mental health problems are described in relevant literature [3,4]. Post-traumatic stress disorder (PTSD), depression, anxiety disorder, somatoform disorders, and substance abuse are the most commonly reported mental health issues [3,5]. These facts result in a high need for psychosocial support services among refugees and asylum seekers which are, however, often insufficient and associated with many barriers [6,7,8,9].

Newly arrived refugees and refugees living in refugee camps are especially vulnerable to mental health problems [10,11]. Cross-sectional studies in different state registration and reception centers in Germany have found PTSD prevalence rates between 23.6% and 40% [5,8,12,13,14]. Still, psychosocial support offers in state registration and reception centers across Germany are sparse, although evidently urgently needed [15,16]. In Germany, clinical care is provided by inpatient and outpatient services. Currently, 26 psychiatric and psychosomatic clinics offer outpatient and inpatient support for migrants and refugees [17]. However, rehabilitation centers for refugees and survivors of torture as well as relief organizations play an important role in mental healthcare for mentally burdened refugees [17]. These rehabilitation centers’ primary services for refugees and torture survivors include psychosocial and psychological counseling, psychotherapeutic sessions, crisis intervention, stabilization work, diagnostics and clearing, and social counseling. According to the annual report of the Federal Association of Rehabilitation Centers for Refugees and Survivors of Torture (Bundesweite Arbeitsgemeinschaft Psychosozialer Zentren für Flüchtlinge und Folteropfer e.V, BAfF), 22,746 refugees and torture survivors received psychosocial care in 2018, of which approximately 41% had psychotherapeutic treatments [18]. Furthermore, various clinical care models exist in the literature. Brakemeier et al. [19] described an interpersonal integrative pilot project for refugees with mental disorders with promising effects in reducing symptoms of PTSD, depression, and anxiety disorder. Other models of care, such as the STEP-by-STEP approach in a German registration and reception center [20], or the Baden-Württemberg humanitarian reception program for Yezidi women and children who have fled captivity of the so-called Islamic State [21], have been reported, but data on their effectiveness have not yet been published. According to Bauhoff and Göpffarth [22], asylum seekers in Germany are twice as likely to report psychiatric hospitalization than regularly insured persons. However, this group also has over three times less access to psychotherapists than regularly insured patients. Satinsky et al. [7] examined the utilization of mental health and psychosocial support services in European countries in their systematic review. They concluded that refugees and asylum seekers are more likely to use medical/somatic health services and to be hospitalized for mental health problems [7]. Providing appropriate care shortly after the refugees’ arrival in the host country can lead to initial stabilization of symptom burden and, thus, prevent further exacerbation and chronification of mental health problems.

Since 2016, a psychosocial walk-in clinic has supported psychologically burdened refugees within the state reception and registration center “Patrick Henry Village” in Heidelberg, Germany. For this center, a mental health inventory has shown that a sample of 228 patients attending the psychosocial walk-in clinic was burdened by PTSD (41.2%), adjustment disorder (22.4%), depression (25.0%), anxiety disorder (6.1%), substance abuse (10.5%), and somatoform disorders (5.3%) [14]. Next to supportive and stabilizing counseling offers, including group psychotherapy [23,24] as well as a program facilitating the self-practice of stabilizing techniques via audio-files [25], half of the patients received psychopharmacological treatment. Outpatient psychotherapy treatment was recommended to 66% of patients after reallocation to municipal housing [14]. Together, these data underscore the necessity of needs-based psychosocial support services shortly after the refugees’ arrival in the host country. So far, the patients’ perspective regarding their experiences with the psychosocial walk-in clinic in a German state registration and reception center has received little attention. Considering the perspective of refugee patients, however, can improve the quality of psychiatric care and provide important implications for future treatment. Carey [26] noted that focusing on patient preferences, needs, and values (as is intended by patient-centered approaches) can help reduce time spent in hospital, readmissions, and emergency room visits, as well as improve compliance and engagement. Considering the patient perspective via qualitative research methods allows us to systematically illustrate and animate individual narratives and, thus, helps us avoid reducing patients to mere diagnoses, numbers, or test subjects [27].

Therefore, this study aimed to gain a deeper understanding of the barriers that refugees face in their mental healthcare efforts to develop strategies in addressing them. To this end, we assessed the perspectives of refugees attending the psychosocial walk-in clinic in the state registration and reception center in Heidelberg, with a focus on the following research questions: (1) How do patients experience their mental health burden and how do they deal with it? (2) What are facilitating and impeding factors when seeking help in the psychosocial walk-in clinic? (3) How do the patients experience the consultations in the psychosocial walk-in clinic? (4) What are the patients’ future attitudes towards further help-seeking behavior?

## 2. Materials and Methods

### 2.1. Data Collection

From March to May 2019, we conducted a descriptive study using qualitative semi-structured interviews in the refugee state registration and reception center ‘Patrick Henry Village’ (PHV), Heidelberg-Kirchheim, Germany. The PHV are former US military barracks currently accommodating around 1,200 newly arrived refugees and asylum seekers. During their PHV stay, newly arriving refugees and asylum seekers’ personal data are registered, their identity is verified, and a medical examination for communicable diseases is carried out as part of the asylum procedure. As a rule, refugees and asylum seekers are redistributed to other accommodations within a short period of time. Since 2016, the Heidelberg University Hospital has been operating a medical and psychosocial walk-in clinic at the PHV in cooperation with physicians in private practice [28,29].

Our target group were refugees who sought help in the psychosocial walk-in clinic [28] and fulfilled our inclusion criteria. Inclusion criteria were an age of 18 or older and the ability to understand one of the following languages: German, English, French, Farsi, Arabic, Turkish, Kurmanji, Urdu, Hausa, Russia, Serbian, Albania, Macedonian, Georgian, Mandinka, or Tigrinya. We asked the refugees to participate in our study while they were waiting for their psychosocial counseling appointment. If a refugee was unable to converse in German or English, a PHV-based interpreter was called or a telephone interpreter was contacted via an interpreter service. If the individual was willing to participate in the study, they were interviewed after their counseling appointment. First, sociodemographic data questions, such as age, nationality, religion, and education level, were answered and then the interview was conducted. The clinical diagnosis information was collected from the participants’ medical files after the consultation appointment. The interviews were conducted by one of the two first authors (V.Z.) who has a medical background.

### 2.2. Participants

In total, *n* = 49 patients waiting for their counseling appointment at the psychosocial outpatient clinic were asked to take part in the study. We interviewed *n* = 22 of 49 patients (44.9%). *n* = 11 (22.4%) patients did not meet our inclusion criteria. Other reasons for non-participation were cognitive impairment (*n* = 3, 6.1%), unwillingness (*n* = 9, 18.3%), or parallel appointments (*n* = 4, 8.2%). Table 1 shows the sample characteristics of the *n* = 22 study participants. *n* = 2 interviews were held in English, *n* = 1 interview was done with a face-to-face interpreter, and *n* = 19 interviews were conducted using a telephone interpreter.

### 2.3. Setting—Psychosocial Walk-In Clinic in the PHV

The psychosocial walk-in clinic is a psychosocial support offer for mentally burdened refugees. Its team consists of six professionals with psychiatric, psychosomatic, and psychotherapeutic expertise working in the Heidelberg University Hospital‘s Department of General Psychiatry, Department of Internal Medicine and Psychosomatics, and the Institute of Medical Psychology [28]. Since June 2019, the psychosocial walk-in clinic offers consultation hours three times a week; previously, the service was offered twice a week. Each week, the clinic can provide counseling sessions to fifteen to twenty refugees. Each consultation session is staffed with two members of the psychosocial walk-in clinic and includes clinical diagnostics, documentation, supportive and stabilizing counseling, psychopharmacological medication, and further treatment recommendations [28,29].

### 2.4. Semi-Structured Qualitative Interviews

We used semi-structured, qualitative interviews to collect data on the interviewees’ experiences of their mental health problems and the counseling services provided at the PHV psychosocial walk-in clinic. The respective interviews were designed based on the methodological approach by Helfferich [30]. The semi-structured interviews comprised key questions which were followed by probing and more detailed clarifying questions. Table A1 shows the interview guideline used for this study.

### 2.5. Quantitative and Qualitative Data Analysis

Demographic variables and baseline characteristics were analyzed using descriptive statistics (frequencies, means, and standard deviations (SD)) and managed with the Statistical Package for the Social Sciences (SPSS) program version 24 [31]. Statements regarding the access routes to the psychosocial walk-in clinic and satisfaction with counseling session were analyzed descriptively (see Table 1).

The qualitative interviews were digitally recorded and transcribed verbatim by one of the first authors (V.Z.) using predefined transcription rules. The qualitative data were analyzed with the software MAXQDA [32] following the principles of qualitative content analysis, as described by Mayring [33]. To do this, we first defined each statement (single or multiple sentences) related to our key questions as a content analytic unit of analysis. Double statements were eradicated, and one statement referred to only one category, so no double coding was possible. We went through each transcribed interview and identified single or multiple content-bearing sentences as quotes, representing the most elemental unit of meaning [34]. Accordingly, these quotes were coded and hereby paraphrased with a term or a short sentence (coding) to summarize them into a relevant category. Thereafter, the categories were grouped into main themes until we could define a number of relevant main themes for all participants. Finally, we discussed the categories and main themes to reach consensus and adjusted them, if necessary [33]. Statements which did not refer to any of our key questions were not analyzed.

## 3. Results of the Qualitative Interviews

We identified 315 statements that were coded and summarized into categories. Finally, eleven categories and four main themes were derived. In the following paragraphs, we will present the main themes and categories. Table A2 shows examples of statements for each individual category within the main themes.

### 3.1. Mental Burden from the Patients’ Perspective

The interviewees described their mental health problems symptomatically, with regard to their illness attributions, and in terms of their perceived future mental well-being.

- **Symptom level (49 quotes):** Interviewees reported psychological and psychosomatic difficulties. The majority of interviewees reported sleep problems, fears, and worries related to family members, being separated from their family, being forced to leave Germany, and the police. They also experienced rumination, decreased well-being, derealization, intrusions, fatigue, and stress symptoms. Interviewees described despairing over severe affective states including agitation, aggression, hopelessness, and loneliness. Less frequently, they also reported other psychosomatic complaints, such as loss of appetite, headaches, and kidney, nose, throat, or heart pain.
- **Disease attribution (43 quotes):** Most of the interviewees attributed their mental burden to past traumatic experiences during flight and/or in their home country. Some specified that they had experienced highly traumatic and stressful events including discrimination, physical abuse, sexual assault, war, torture, loss of family members, as well as the loss of their homes and previous communities. In addition to past events, the psychological burden was considered to be linked to their current situation, including uncertainty about the asylum process, anxiety and apprehension regarding deportation, the future, and the well-being of family members. Most notably, interviewees often highlighted their current living conditions as a major exacerbating contributor to their psychological burden. For example, one participant stated that he no longer felt like a normal human being and that the accommodations were demeaning.
- **Coping strategies for mental burden (35 quotes):** The majority of interviewees felt that social support from their family and/or friends helped them cope with their psychological symptoms. One participant said that being around friends or acquaintances helped him forget about his mental burden, while another participant felt that talking to somebody and receiving advice helped him. Other interviewees specifically named emotional coping strategies, such as allowing themselves to feel emotions, letting go of existing fears, and finding hope or a sense of security. Several interviewees mentioned activities or behavioral strategies that (may) help them cope with their mental burden, such as attending a language class, participating in the stabilization group offered at PHV, or engaging in physical activity. Some of the interviewees reported considering professional support, like medical or therapeutic care, as an important part of their coping strategy. One interviewee said that practicing his religion helped him to cope with his symptoms. Interviewees were also asked how they would deal with symptoms in their home country. About half of the interviewees reported that they would have reached out for medical or therapeutic support. In contrast, others indicated that they would not have had the possibility of getting help back home. Several interviewees said that their social network and community had been their main coping strategy. One participant said that he thought he would have become suicidal if he had remained in his country.
- **Expected future course of mental health (24 quotes):** The majority of the interviewees felt optimistic about their future mental health. One participant said that she felt optimistic about getting better because her family (husband, children) were now in safety. However, the interviewees also emphasized several necessary prerequisites before they felt their mental health could improve, including feeling safe, being less exposed to noise in the center, receiving medical and psychological support, and being granted asylum in Germany. One person stated that she thought she would feel better if she could find a goal for her future. Others thought that they would get better if they were able to receive regular medical and therapeutic help in the future. Some interviewees said that they hoped that they would feel better next year, while others said they were unable tell. One patient expected his mental health to deteriorate further in the future.

### 3.2. Access to the Psychosocial Walk-in Clinic in the PHV

Regarding the contact to the psychosocial walk-in clinic, the interviewees described the following impeding and facilitating factors:

- **Barriers (25 quotes):** Most interviewees did not mention any impeding factors. One interviewee stated that if he started something he finished it. Some interviewees reported structural barriers to treatment, and insufficient counseling appointments to meet existing needs and the resulting long waiting times were mentioned most frequently. One participant said that he had been unable to get an appointment the preceding week. Personal barriers were rarely mentioned, but often included stigmatization fears and feelings of shame about seeking psychotherapeutic support. One interviewee stated that he had been quite nervous about the upcoming appointment.
- **Facilitating factors (12 quotes):** Interviewees reported that most often, medical staff, social legal process counseling staff, PHV interpreters, and other interviewees at the psychosocial outpatient clinic had encouraged them to use the therapeutic services. Others said their faith in the effectiveness of therapy had motivated them to seek help at the clinic. One participant stated that he had prior therapy experience. The interviewees highlighted the clinics’ walk-in approach and relatively short waiting times as key facilitating structural aspects.

### 3.3. Perception of Counseling Sessions

The interviewees named helpful and difficult aspects during counseling interactions.

- **Helpful (37 quotes):** Most interviewees experienced the counseling sessions as helpful. Specifically, the interviewees said that they gave them confidence, encouragement, hope, and orientation. Other interviewees said that the conversations with the therapists soothed them or made them feel better. Several interviewees mentioned that they felt that the therapists’ attentive, respectful, and caring attitude particularly helped them. One participant stated that she appreciated that the therapist had not asked her about her failed suicide attempt in too much detail. Other interviewees reported experiencing feelings of safety and trust because of the counseling interactions. For example, one participant stated that he felt the therapist trusted and cared about him. Interviewees frequently named building a trustful relationship with the therapist as well as receiving psychiatric medication as the most helpful supporting factors in their experience of the walk-in clinic. In addition, interviewees appreciated learning stabilization techniques in the PHV’s group therapy services and receiving medical reports.
- **Difficulties (42 quotes):** Most interviewees stated that they had not experienced anything difficult or strange during the counseling sessions. One participant stated that nothing had been able to help him yet. A few interviewees mentioned structural difficulties, such as crowded waiting areas, hearing other patients during counseling sessions, and interpreter-mediated communication. Personal difficulties included finding it stressful or upsetting to talk about certain experiences or, conversely, not being able to specifically address certain topics. One interviewee felt that the different cultural backgrounds also impeded the patient–therapist alignment and mutual understanding.

### 3.4. Perception of Follow-Up Treatment

The interviewees also described their motivation as well as facilitating and impeding factors regarding further therapeutic help outside the state registration and reception center following their move to municipal housing.

- **Motivation (30 quotes):** Most interviewees stated that they would like to continue receiving therapeutic support in the future. Other patients answered that they would do so depending on how their psychological complaints would develop or whether the social environment was in favor of further treatment. Three interviewees indicated that they were unlikely to seek further treatment because they believed that a secure residence status in Germany would necessarily lead to an improvement of their mental state.
- **Barriers (11 quotes):** Interviewees mainly listed internal barriers preventing them from seeking mental health services in the future, including feelings of shame about seeking therapeutic support or having mental health problems, fear of stigmatization, as well as memory- and concentration-related difficulties. For example, one participant said that she was worried that peers might ridicule her, if she went to school and they found out she was seeing a therapist and taking medication. They also mentioned structural problems, which included lack of time, insufficient language skills, difficulties in obtaining information about therapeutic services, and a negative asylum decision.
- **Facilitating factors (7 quotes):** Interviewees felt that the widespread availability of mental healthcare services in Germany along with ample opportunities to find out about them would make it easier for them to find follow-up psychosocial treatment offers. One participant stated that there were laws in Germany to this regard. Additionally, interviewees stated that they felt they could access follow-up treatment with the support of family or friends.

## 4. Discussion

This study aimed to shed light on refugee patients’ perspectives on their psychological burden, use of low-threshold healthcare, as provided by the psychosocial walk-in clinic in a state registration and reception center in Germany, and their future help-seeking attempts regarding follow-up treatments. Our qualitative results show that psychological stress was mainly described on an emotional and cognitive level and less frequently voiced via psychosomatic symptoms. Additionally, participants emphasized the impact of post-migratory stressors as exacerbating contributors to their mental health problems. The majority of interviewees were satisfied with the counseling provided by the psychosocial walk-in clinic. Participants particularly appreciated the supportive, resource-focused, and trust-building conversations with the therapists, pharmacological treatment, as well as access to ancillary services, such as a stabilization group, and the receipt of medical reports. Prior recommendation and encouragement by others as well as the belief in the effectiveness of therapeutic support were named as factors facilitating psychosocial walk-in clinic attendance. Perceived difficulties were mainly seen in structural barriers, such as the clinic’s confined waiting area and the generally high noise level, as well as long waiting periods. However, interviewees also named internal factors, like feelings of shame and fear of stigmatization. Most interviewees felt motivated to seek further therapeutic support in the future. Regarding barriers to future therapeutic support, participants named internal difficulties, such as feelings of shame, fear of stigmatization, as well as language barrier-related concerns and lack of information about respective support offers.

Regarding their mental health, the interviewees most frequently stated psychological problems on an emotional and cognitive level and only named a few somatic symptoms. While several previous studies have suggested that refugees tend to express their psychological distress through somatization [35,36,37], statements regarding somatic complaints were less frequent than one might have expected in this study’s sample. The symptoms described by the respondents can be seen as part of their mental illnesses, such as PTSD, depression, and adjustment disorder. Especially somatic symptoms, like heart pain or heart excitement (see Reference [36]), can often be somatic manifestations of the patient’s world of affective experience. Our data suggests that our interviewees had a high awareness of mental illness as well as good symbolization abilities and did not seem impeded by the various reasons previously named to explain high somatization rates in refugee populations, which include social and cultural acceptability, fear of stigma, somatic rather than psychological symptom expression, somatization as a cultural sign of distress, and alexithymia [38]. However, our interviewees’ good symbolization abilities, as in their ability of expressing emotions and understanding their somatic manifestations, might be explained by the fact that they were patients attending the psychosocial walk-in clinic for mentally burdened refugees and asylum seekers and, thus, are a rather more select group.

The burdening symptoms were attributed to past events and to post-migratory distress factors. This is in line with previous findings in which refugees have named past and flight-related experiences as well as stressful aspects of the current life situation as causes for their poor mental health [35,39,40,41,42]. For instance, in Zbidat et al.’s study [36], symptoms of insomnia or fatigue were linked to events like loss of one’s family, possessions, and home. Further, post-migratory distress factors were associated with enhanced vulnerability for mental health problems [43,44,45]. Interestingly, previous studies have also identified supernatural and religious beliefs as important factors in their participants’ theory of illness regarding their mental illness [46,47]. Spiritual or traditional belief systems regarding the causes of psychological symptoms may be associated with help-seeking behavior outside the healthcare system [48]. However, none of the interviewees mentioned any religious or supernatural factors in our study sample. This may be explained by the fact that mental health literacy increases with the use of mental health services [49,50,51].

In addition to psychological burden, interviewees reported various coping strategies, including social support or emotional and behavioral strategies. Religious practice was only specifically mentioned once. Social support and religion have been named as key and preferred coping resources in previous studies, while seeking professional help was rare [39,40]. Interestingly, consulting a therapist/medical professional was a frequently cited coping strategy in our study. This could be explained by the fact that the psychosocial walk-in clinic is an established and well-accepted service in the registration and reception center. This may not only facilitate low-threshold healthcare access but also promote mental healthcare awareness as well as acceptance. Furthermore, the interviewees in our sample seemed to be rather open-minded towards professional medical and therapeutic help. This is reflected by the fact that 50% of the interviewees said they would have reached out for a professional in their home country. The fact that professional help was seen as so important may also be related to the fact that the other coping strategies alone were no longer experienced as sufficient to manage the increasing psychological symptoms. Especially, the early post-migratory phase holds multiple challenges, such as uncertainty about the asylum procedure, frequent reallocation to other accommodations, and having to start adjusting to a new country and a new environment. This can cause trusted coping skills to become inadequate.

Interviewees were hopeful about the prospective course of their mental state. Indeed, the majority expected to see improvement in the future. This is particularly interesting considering that negative cognitions and emotions, such as hopelessness, helplessness, and resignation, are frequently associated with the diagnoses of post-traumatic stress disorder, depression, and adjustment disorders [52]. Keeping in mind that optimism can impact mental well-being, this optimistic attitude is a valuable resource [45]. However, different longitudinal studies underline that mental healthcare needs are extremely high among refugees and asylum seekers [10,53,54,55]. For instance, Nikendei et al. [14] conducted a three-month follow-up study to assess patients’ further course of mental health after attending the psychosocial walk-in clinic in the PHV. While they were able to show improvement in depression, panic, and psychosocial well-being, the levels were still clinically relevant. No changes were found for PTSD or generalized anxiety disorder. They further examined access to healthcare and concluded that while most patients had access to general practitioners and local psychiatrists, none of the assessed refugees had access to outpatient psychotherapy. Regarding the access to the psychosocial walk-in clinic, facilitating factors, such as recommendation and encouragement by medical or social staff members working in the PHV as well as other refugees, helped them reach out to the psychosocial walk-in clinic. Only a few interviewees came on their own accord. According to Asgary and Segar [56], stigmatization, shame, mistrust, low trust in mental health services, lack of information, and low health literacy are key internal impeding factors in mental healthcare access. Personal and professional referrals appear to raise awareness, motivate, and encourage affected individuals and reduce initial fears of stigmatization, not least by providing necessary information. Our findings suggest that the interplay of different mental health and medical organizations is of great importance to facilitate refugees’ access to mental health services, especially with regards to overcoming barriers. Unfortunately, difficulties in recognizing and dealing with clinical and social problems, low between-healthcare provider inter-collaboration, as well as diagnostic insecurities often impede professional help [6,7]. In the present study, interviewees mentioned few barriers preventing them from attending the walk-in clinic. This may be explained by the clinic’s low-threshold service structure characterized by easy accessibility, walk-in policy (no prior appointment needed), free treatment, and on-site interpreters.

Most interviewees were highly appreciative of the counseling sessions and felt that the conversations there helped them. In the literature, conveying hope and confidence, feelings of safety, trust, confidentiality, as well as respectful, appreciative encounters, form the foundations of positive therapeutic interactions [57,58,59]. Several authors argue that the importance of these relational aspects becomes even more significant due to the backdrop of the adverse and dehumanizing experiences refugees have suffered [58,60]. Accordingly, psychosocial work with refugees should be directed toward a therapeutic relationship that is conducive to trauma management. This is, in fact, very appreciative of the professionals working in the psychosocial walk-in clinic. Particularly, since counseling sessions in the clinic are often the refugees’ first experience of therapy, and expectations differ due to difficulties in distinguishing between the professional focus of psychologists, psychotherapists, psychiatrists, and general practitioners.

While the positive perception of psychopharmacological treatment found in our study is consistent with previous findings [61], biological components of mental health disorders were not explicitly represented in their theories of their mental illness. Frequently, inadequate mediation intake or low compliance can be a problem [62]. According to Nikendei et al.’s [14] follow-up study, 51.9% of patients had continued taking their prescribed medication three months after they had last visited the psychosocial walk-in clinic. Interestingly, medication was one of the refugees’ least preferred coping strategies in Markova et al.’s [40] study. Here, older participants in particular were more skeptical towards psychopharmacotherapy. However, in our study, the respondents were around thirty years old. Consequently, one could speculate that they might have been more receptive of medication and more open-minded in their help-seeking behaviors. Nevertheless, the interviewees also valued the stabilization-focused group psychotherapy as an adjacent offer. Stabilization group psychotherapy [23,24] and the self-practice of stabilizing techniques via audio-files [25] have been shown to increase mentally burdened refugees’ emotional stability in the early post-migratory phase. In general, guided-imagery techniques can be a valuable resource in cross-cultural work and treating PTSD [63,64].

Difficulties in the context of therapeutic encounters can include the use of interpreters [65,66], fear of verbalizing (emotional) problems [67], and differences due to cultural differences [68]. Furthermore, doubts about the usefulness or effectiveness of psychosocial treatment have also been addressed in previous studies [58,67]. Although perceived problems matched the inhibiting factors described above, they seemed to be low in our study, where feelings of gratitude for receiving therapeutic support clearly outweighed perceptions of difficulties. Unfortunately, interculturally trained, foreign language, and specialist language qualified therapists are rare and mental health services often have to rely on interpreter services. Still, the patient’s and the interpreter’s cultural background as well as their fit should always be taken into account. Professional knowledge about cultures and culturally sensitive communication are key in efforts toward bridging cultural barriers in healthcare. Hence, culturally sensitive communication training programs should be established. Regarding the high levels of noise in the waiting area, a respondent advised putting up “Please be quiet” signs, which could prove to be a simple solution to a big problem. However, as the clinic location is in former military barracks, which were by no means originally built for medical and psychosocial care, these structural barriers will be difficult to solve and reflect the difficult accommodation situation that refugees face in the PHV. In addition, it must be noted that questions about critical aspects or suggestions for improvement often remained unanswered or received evasive answers.

With regard to the pursuit of follow-up treatment outside of the PHV, most patients felt motivated to reach out to psychosocial services after municipal accommodation. Nonetheless, some patients hoped that their mental health would improve as post-migratory stressors subsided and felt they would not need further help. In line with previous findings [56,59,60,69], our sample cited shame, fear of stigmatization, lack of time, symptom-related difficulties, and lack of information as barriers. Overall, patients were optimistic about their post-reception center treatment access possibilities to therapeutic care. However, despite the refugees’ belief in the availability of psychosocial care structures, there is still a great shortage of psychosocial care services in reality [16,17]. While the psychosocial walk-in clinic within the center is undoubtedly an important service, such offers are regrettably rare. Rehabilitation centers for refugees and survivors of torture as well as relief organizations play an important role in later mental healthcare for mentally burdened refugees in Germany [17]. Still, the attendees’ positive experiences during therapeutic consultations in the walk-in clinic may help refugees later when seeking further psychosocial support. Strengthening the interconnections between the diverse actors involved in the psychosocial care of refugees is essential to ensuring that transitions to further direly needed treatment are successful. Patients attending the psychosocial walk-in clinic receive a medical report which includes information about their reason for attendance, symptoms assessed during the counseling session, clinical diagnoses, medication prescriptions, and further healthcare referrals. This can provide future healthcare professionals with an impression and guidance as to how to proceed. Unfortunately, interconnections between the psychosocial walk-in clinic and local professionals are still limited. Furthermore, as refugees are often relocated all over the country after leaving the intimal reception center, a nationwide network needs to be established.

## 5. Limitations

This qualitative study has several limitations. First, it relies on self-reports as is common in qualitative research. Hence, we cannot rule out compliant or socially desirable responses. We conducted the interviews shortly after a counseling session. Hence, the interviewees may have responded in favor of the psychosocial walk-in clinic. Second, we did not examine possible culture-specific influences which may have affected our results. Cultures differ regarding gender roles [70]; therefore, it has been suggested that the professional’s ethnic background and/or gender should match the patient in professional mental health settings [71]. In our study, the interviewer was female which might have affected the interviewees’ responses. Further, most interviews were conducted using a telephone interpreter with whom we had no prior contact. Colucci et al. [71] pointed out that gender, age, as well as cultural and ethnic dynamics should be considered when using an interpreter. Hence, the interviewees’ responses may also have been influenced by the interpreter as we were unable to match their cultural backgrounds. Third, our analysis followed Mayring’s principles of the qualitative content analysis [33]. Accordingly, content-analytical analysis units were defined before the qualitative analysis. In our study, units were defined as any statement (single or multiple sentences) referring to our key questions. This procedure may have led us to overlook other emerging themes in the interviews which were not part of the key questions. Fourth, limiting our results’ generalizability, the psychosocial walk-in clinic’s patients are a specific and selected group of refugees seeking help. Furthermore, we did not assess their mental health history in their country of origin or specifically record if the symptoms had occurred due to pre- and/or peri-migratory distress factors. Refugees and asylum seekers fleeing their country of origin because of limited access to mental health services are likely to be more open to mental health services in the host country.

## 6. Conclusions

The psychosocial walk-in clinic within the registration and reception center is perceived as an important psychosocial support offer for mentally burdened refugees in their early post-migratory distress phase. Current living conditions and post-migratory distress factors were stated as particularly burdening. The interviewees saw the therapists’ attitude as a very important factor. Further training focusing on cultural differences and cultural-sensitive communication could be installed to improve the offer. The refugees’ positive experience of the psychosocial walk-in clinic may help them overcome internal barriers when seeking mental health treatment in the future. Nevertheless, mental healthcare literacy programs and nationwide interconnections between professionals are crucial in providing refugees with adequate access to mental healthcare offers.

## Figures and Tables

**Table 1:** Sociodemographic sample characteristics.

Sample Characteristics (*n* = 22)	
	*n* (*%*)
Gender	
Female	10 (45.5%)
Male	12 (54.5%)
Years of education	
<10 years	13 (59.1%)
>10 years	5 (22.7%)
University degree	4 (18.2%)
Education	
No education	7 (31.8%)
Professional training	9 (40.9%)
Academic education	4 (18.2%)
No data	2 (9.1%)
Country of origin	
Eastern Europe	5 (22.7%)
Asia	12 (54.5%)
Africa	5 (22.7%)
Religion	
Christianity	6 (27.3%)
Islam	13 (59.1%)
Judaism	2 (9.1%)
Atheism	1 (4.5%)
Relationship status	
Single	9 (40.9%)
Married	10 (45.5%)
Divorced	1 (4.5%)
Partnership	1 (4.5%)
No data	1 (4.5%)
Access routes to the psychosocial walk-in clinic ^a^	
Self-initiated	6 (27.3%)
Other refugees	3 (13.5%)
Court order	1 (4.5%)
Physician referral	7 (31.8%)
Counseling center	4 (18.2%)
Not specified	1 (4.5%)
Satisfaction with counseling session ^a^	
Satisfied	13 (59.1%)
Not satisfied	3 (13.5%)
Not specified	2 (9.1%)
Diagnoses	
PTSD	14 (64.0%)
Depression/adaptation disorder	17 (77.0%)
Both diagnoses	9 (41.0%)
	M (SD); Range
Age (Years)	32.95 (12.06); 18–57
Number of psychiatric diagnosis	1.47 (0.70); 1–3
Number of children	1.55 (2.06); 0–9

Note: ^a^ information provided by the semi-structured interviews. PTSD: post-traumatic stress disorder. M: mean, SD: standard deviation.

## Data Availability

The datasets used and analyzed during the current study are available from the corresponding author on reasonable request.

## Appendix A

**Table A1:** The Interview Guide Used for the Current Study.

Interview Guide
• How did you hear about the Psychosocial Walk-In Clinic in the PHV?
• What made it difficult to use this offer?
• What were your reasons for using the offer?
• What was helpful?
• What was not helpful/difficult for you during the consultation?
• What was strange/difficult before? Was something different than you expected?
• What else would you have needed during your consultation?
• Did you get the support you hoped for during the consultation?
• Which complaints are currently bothering you?
• Where do you think your current complaints come from?
• How do you deal with your complaints?
• How would you deal with such complaints in your home country?
• How will your symptoms change next year?
• What is your opinion on psychological support as a medical treatment method in general?
• Would you like to receive psychosocial support after your stay in PHV?
• Do you think you will seek further psychotherapeutic help after the PHV?

**Table A2:** Examples of Statements for Each Individual Category within the Main Themes.

Main Themes and Categories	Example Codes
(A) Mental burden from the patients’ perspective	
• Symptom level	*I want to rest, I want to be better, I used to be a coach, I want to be very well. It was different before I was doing very well. Now, I am exhausted. I don’t sleep, I don’t eat, I started smoking again.*
	*So doctor…, I also usually have heart pains when… I remember earlier or all the memories come up, then, I also usually have heart pains and then I feel very bad.*
• Disease attribution	*This whole thing with what he, what he’s been through, that makes him mad.… the things, the stories, what’s happened so far, and the past makes me sad.*
	*So, I am stressed because of that, quite simply, on the escape route, on our escape route in Greece, there (…) there was a knife attack on the husband of my girlfriend who was traveling with us and from that moment on I was afraid*
	*Ninety percent of my fears… so those are my return to Italy*
	*Now, he is here, and he has expanded his life again more or less. He has found friends; he has found rituals in his life … The, he’s definitely tried to make his environment as familiar to him and as he feels comfortable. Now, when he thinks that he would have to leave that again and start somewhere else, something new again, that gets him down.*
• Coping strategies for mental burden	*When I am with the friends, acquaintances,… then I forget my,…problems. Then I feel a little… better*
	*If I cried now… so that I would somehow be more relieved then maybe it would get better*
	*It would help me a lot if I could go to school. Because I was such a good student. And I love going to school (laughs). Many, many here don’t like it. But I really do.*
	*It helps him… that he is with the doctor. (…) And he also knows that everything takes time.*
• Expected future course of mental health	*I hope so and I think so, right or not.*
	*It all depends. I think when I find a way and have a goal of what I’m going to do next, I feel better. But without the goal and the plan what will become of me then I feel bad. This uncertainty of what’s going to happen to you.*
	*It will be better for me here in Germany, I think. Things will be a little bit better. Yeah. Even not with money or anything but with my life. Yeah. I think it will be better here*
(B) Access to the psychosocial walk-in clinic in PHV	
• Barriers	*Only the difficult thing is, to meet him, because it is from Monday in the morning and Wednesday in the afternoon. And sometimes, if you come in the afternoon late, you could not meet him because many people are there.*
	*Yes,… I was kind of afraid that people would find out and then laugh at me because I’m here with a psychologist now (yes). Yes.*
• Facilitating factors	*Yes, I, I thought I (..) I will be helped. I will be able to sleep better, feel better.*
	*So he got that from his interpreter.*
	*Without making… an appointment that is a relief for us to just come by and get treated. That is a great relief for us.*
(C) Perception of counseling sessions	
• Helpful	*Yeah, she encouraged me and listened to me. I have everything is going to be okay* *They have motivated me. You have made me brave.*
	*I also liked the way they… which medications I need,… I also liked that.*
	*And the exercises that in some situations where I have so quite stress that I calm down a bit.*
	*They have applied to the social welfare office and the court that I am allowed to go to my family.*
• Difficulties	*No, nothing seemed strange to us, completely normal, just like us.*
	*He has difficulties just talking about it, so he wants to say it but he realizes (…) it’s hard for him. So he finds it hard to talk about it in general.*
	*…he says since we have cultural differences, it would be much better that he goes to a doctor, for example, that he has Persian background. And he can understand him better. So now with the translator and the language and then the different questions, which for him sometimes also has no sense, because it just does not fit to his culture*
	*The noise that she actually hears while she’s sort of in this consultation, that sort of messes her up sometimes.*
(D) Perception of follow-up treatment	
• Motivation	*I would like to, because I think it is very important and it affects many African people. They don’t know this. Yeah. I never been to a psychiatric doctor before in my life. So this is important. And I will be going all the time.*
	*If I would continue to have my problems, then I will…. see a psychologist*
	*No. I hope God is the one who gives health.*
• Barriers	*Only that others find out and then laugh at me.… Because I’ve been bullied so often, I’m really so afraid of it (laughs).* *He just doesn’t know because he can neither speak German nor English how he will get to it later.*
• Facilitating factors	*If we were allowed to stay in Germany or were not deported, we could make use of all (.) help. There are enough human rights in Germany in this regard.*
